# Non-alcoholic fatty liver disease induces signs of Alzheimer’s disease (AD) in wild-type mice and accelerates pathological signs of AD in an AD model

**DOI:** 10.1186/s12974-015-0467-5

**Published:** 2016-01-05

**Authors:** Do-Geun Kim, Antje Krenz, Leon E. Toussaint, Kirk J. Maurer, Sudie-Ann Robinson, Angela Yan, Luisa Torres, Margaret S. Bynoe

**Affiliations:** Department of Microbiology and Immunology, College of Veterinary Medicine, Cornell University, Ithaca, NY 14853 USA; Department of Biomedical Sciences, College of Veterinary Medicine, Cornell University, Ithaca, NY USA; Center for Animal Resources and Education, College of Veterinary Medicine, Cornell University, Ithaca, NY USA; Center for Comparative Medicine and Research, Dartmouth College, 1 Medical Center Drive, 302 W Borwell, Lebanon, NH 03756 USA

**Keywords:** Alzheimer’s disease, Apolipoprotein E, High-fat diet, Non-alcoholic fatty liver disease, Standard diet

## Abstract

**Background:**

Non-alcoholic fatty liver disease (NAFLD) is a chronic liver disease afflicting about one third of the world’s population and 30 % of the US population. It is induced by consumption of high-lipid diets and is characterized by liver inflammation and subsequent liver pathology. Obesity and consumption of a high-fat diet are known to increase the risk of Alzheimer’s disease (AD). Here, we investigated NAFLD-induced liver inflammation in the pathogenesis of AD.

**Methods:**

WT and APP-Tg mice were fed with a standard diet (SD) or a high-fat diet (HFD) for 2, 5 months, or 1 year to induce NAFLD. Another set of APP-Tg mice were removed from HFD after 2 months and put back on SD for 3 months.

**Results:**

During acute phase NAFLD, WT and APP-Tg mice developed significant liver inflammation and pathology that coincided with increased numbers of activated microglial cells in the brain, increased inflammatory cytokine profile, and increased expression of toll-like receptors. Chronic NAFLD induced advanced pathological signs of AD in both WT and APP-Tg mice, and also induced neuronal apoptosis. We observed decreased brain expression of low-density lipoprotein receptor-related protein-1 (LRP-1) which is involved in β-amyloid clearance, in both WT and APP-Tg mice after ongoing administration of the HFD. LRP-1 expression correlated with advanced signs of AD over the course of chronic NAFLD. Removal of mice from HFD during acute NAFLD reversed liver pathology, decreased signs of activated microglial cells and neuro-inflammation, and decreased β-amyloid plaque load.

**Conclusions:**

Our findings indicate that chronic inflammation induced outside the brain is sufficient to induce neurodegeneration in the absence of genetic predisposition.

**Electronic supplementary material:**

The online version of this article (doi:10.1186/s12974-015-0467-5) contains supplementary material, which is available to authorized users.

## Background

Alzheimer’s disease (AD) is a progressive neurodegenerative disease associated with decline in cognitive function, impairment in memory, language and visual-spatial coordination, eventually resulting in complete loss of basic function [1]. Dementia of all forms affects about 5 % of the population older than 65. There are approximately 5.5 million cases of AD in the USA alone, and this number is estimated to nearly triple by the year 2040. Moreover, as the world population lives longer, AD and dementia are predicted to constitute a major global health problem in the aging population of the world [2].

Major pathological hallmarks of AD present as senile amyloid plaques that are composed of β-amyloid (Aβ) protein and intracellular neurofibrillary tangles with characteristic reactive microgliosis and astrogliosis. AD is characterized by dystrophic neuritis, neuronal loss, synaptic dysfunction, and cerebral atrophy [1]. AD can be largely divided into early and late onset forms. Early onset AD is induced in patients carrying genetic mutations in amyloid precursor protein (APP) and/or presenilin (PS1 or PS2) which induces formation of insoluble Aβ. Previous work showed that mice expressing mutated *APP* display alterations in exploratory activity as well as elevation of β-amyloid production reminiscent of AD [3, 4]. Late onset AD, also known as sporadic AD, is believed to be induced by aberrant processing of Aβ resulting in pathological lesions. In general, APP is cleaved by α-secretase which generates soluble Aβ which has neuroprotective functions [5]. However, when APP is cleaved by β-secretase-1 (BACE1), it produces Aβ_1–42_ which is insoluble and forms amyloid plaques that are pro-apoptotic and neurodegenerative and are believed to induce cognitive impairment [6, 7]. While the underlying cause of AD is not known, advancing age, environmental stressors, and genetic factors appear to be important precursors [8, 9]. In addition to Aβ deposits, AD is characterized by neurofibrillary tangles which are neuronal deposits of hyperphosphorylated-Tau, also referred to as tauopathies, which are well correlated with cognitive impairment and advanced neurodegeneration [1, 10]. Due to this association, it is still debated whether the initiating factor of AD is Aβ plaques or tauopathy [11].

In the USA, diets high in fats/lipids, which are commonly known as “fast foods,” are prevalent in a significant portion of the population and are becoming an important public health issue. High-fat diets are implicated in various metabolic syndromes leading to obesity, atherosclerosis, insulin resistance, dementia, cognitive decline, and potentially, AD [12–16]. HFD also induces a liver pathology called non-alcoholic fatty liver disease (NAFLD) which is characterized by fatty liver, accumulation of lipids in hepatocytes, infiltration of inflammatory immune cells in the liver parenchyma, and secretion of pro-inflammatory cytokines resulting in liver damage [13, 14, 17, 18]. NAFLD is the fourth leading cause of liver disease in the Western hemisphere. It afflicts about 30 % of the US population and is the 12th leading cause of death in the USA among adults 45–54 years [19]. The increase in NAFLD has been linked to increased prevalence of obesity and metabolic diseases in the USA and worldwide [7]. NAFLD is associated with marked progressive inflammation, fat deposition, and fibrosis of the liver [8, 10]. Also, a clear association exists between cardiovascular risk factors or carotid atherosclerosis and dementia progression leading to AD [20].

Cholesterol is an important building block of the brain [21], which produces over 20 % of total cholesterol in the body. This high-cholesterol content is needed for neuronal function, as the brain cannot access plasma cholesterol due to restrictions posed by the blood brain barrier [22]. For this reason, neuronal cells express high levels of cholesterol-uptake receptors such as low-density lipoprotein receptor (LDLR), low-density lipoprotein receptor-related protein 1 (LRP1), and apoliprotein-E (ApoE). Up to 70 % of the brain’s cholesterol make up the myelin sheath of oligodendrocytes and the membrane of astrocytes, with the remainder contributing to neuronal function including the myelin sheath of neurons that relay synaptic signals. ApoE and LRP1 are related to cholesterol metabolism and are important risk factors contributing to the prevalence of AD [23–25]. The ApoE variant, ApoE4, increases AD risk and accelerates AD onset (Bu, 2009; Liu et al., 2013). Multivariate analysis of metabolites in the blood of AD patients showed that of the ten metabolites that distinguished AD from its age-matched cohorts, six were long-chain cholesteryl esters that were reduced in AD [26]. Also, several large cohort studies showed that long-term treatment with statins, which lowers serum cholesterol levels, could alleviate AD symptoms, suggesting that alteration in lipid metabolism contributes to AD pathogenesis [27, 28]. LRP1 is an endocytic receptor highly expressed in the liver, on neurons, and on vascular smooth muscle and glial cells in the CNS vasculature and functions in the clearance of Aβ from the CNS [29]. Aβ clearance is impaired in neurons from LRP1-deficient mice [30]. Binding of APP to LRP1 results in increased trafficking and clearance of APP. However, LRP1 is also involved in Aβ production [31]. Hence, its involvement in Aβ synthesis and clearance makes it a prime target in AD pathogenesis. Deletion of LRP1 exacerbated Aβ deposition and increased CAA [29]. It is speculated that ApoE may inhibit or facilitate LRP1 endocytosis of Aβ. Since ApoE4 is linked to both sporadic and familial AD and ApoE functions in the cellular transfer of lipids through LRP1 on the cell surface, it is presumed that LRP1, ApoE, or both are involved in dysfunction of the lipid transport mechanism and Aβ clearance.

The increase in obesity and NAFLD prevalence in our society, both induced by diets high in fats/lipids and their link to chronic inflammation and metabolic diseases, mirrors increase in AD and AD-like syndromes. We therefore decided to investigate the impact of NAFLD in AD pathogenesis in an AD transgenic mouse model (APP-Tg) and in C57BL/6, wild-type (WT) mice.

To elucidate the effect of a diet with increased lipids on AD pathogenicity, we fed WT and APP-Tg mice with a high-fat diet (HFD) or standard diet (SD) for 2, 5 months, or 1 year. HFD induced systemic and CNS inflammation that accelerated Aβ plaque deposition during acute NAFLD (2 and 5 months) in APP-Tg mice. In WT mice, acute NAFLD induced neuro-inflammation but did not induce Aβ plaques. Removal of HFD after 2 months decreased Aβ plaque load in APP-Tg mice and reversed signs of systemic and CNS inflammation in both WT and APP-Tg mice. WT and APP-Tg mice were kept on HFD for up to 1 year to determine the impact of chronic NAFLD in AD induction in WT mice and AD progression in APP-Tg mice. We observed advanced signs of AD, including accelerated cerebral amyloid angiopathy (CAA), increased tauopathy, and increased neuronal loss in APP-Tg mice. More importantly, long-term HFD treatment induced plaque formation, CAA, and tauopathy in WT mice. The advanced signs of AD were associated with a decrease in CNS expression of LRP1 during chronic disease. These studies indicate that HFD-induced inflammation plus aging are sufficient to trigger neurodegeneration and accelerate the process of AD even in the absence of genetic predisposition.

## Methods

### Mice and diet

The APP-Tg mouse [B6.Cg- Tg (APPswe, PSEN1dE9)85Dbo/J] was generated as previously described [32]. WT mice and their APP-Tg littermates were fed either a standard diet (SD) (Harlan Teklad TD.7912) or a high-fat diet (HFD) (1.0 % cholesterol, 0.5 % cholic acid, 18 % triglyceride; Harlan Teklad TD.88051, “Paigen diet”) [14] beginning at the age of 2 months. WT and APP-Tg mice were fed with SD or HFD for 2, 5 months, or 1 year. Another set of APP-Tg mice were removed from HFD after 2 months and were put back on SD for 3 months. All animal work was done in accordance with PHS guidelines and was approved by Cornell’s Institutional Animal Care and Use Committee (Protocol # 2008–0092).

### Tissue harvest

Deeply anesthetized mice were weighed and transcardially perfused with ice-cold phosphate buffered saline (PBS); then, brain, spleen, and liver were collected for analysis. After macroscopic photo documentation, all livers were weighed and used for leukocyte preparation, except two 30-mg tissue sections which were used for histopathology and RNA preparation. Approximately 30 mg of liver and one brain hemisphere were flash frozen in Tissue-Tek O.C.T. (Sakura Finetek) and stored at −80 °C. 10-μm thick frozen sections were affixed to Superfrost/Plus slides (Fisher), fixed in acetone, and stored at −80 °C.

### Immunohistochemistry

For immunohistochemistry staining, slides were thawed and treated with 0.03 % H_2_O_2_ in PBS to block endogenous peroxidase or fixed and permeabilized in acetone for immunofluorescence staining, blocked with casein (Vector Laboratories) in normal goat serum (Zymed), and then incubated with anti-CD45, phospho-Tau, ApoE, CD31, LRP-1, 6E10, GFAP, or NeuN primary antibodies. For immunohistochemistry, slides were then incubated with biotinylated goat anti-rat Ig (Jackson ImmunoResearch) and streptavidin-HRP (Zymed) and developed with an AEC (Red) substrate kit (Zymed), counter-stained with hematoxylin and mounted with Fluoromount-G. For immunofluorescence assay, slides were instead subjected to AF488, TexRed, or AF647 conjugated secondary antibody and coverslips were mounted with Vectastain containing DAPI (Vectorlabs). Standard or frozen histological tissue sections were formalin-fixed and processed for hematoxylin and eosin (H&E) or oil red-O staining and hematoxylin counterstaining, respectively, then examined by light microscopy. For the green fluorescent Thioflavine S (ThioS) staining of plaques, frozen sections were incubated with 1 % ThioS (Sigma-Aldrich) in distilled water for 5 min, differentiated in 70 % ethanol for 5 min, washed three times for 5 min each with distilled water and cover-slipped with Vectastain containing DAPI (Vectorlabs). Images were captured using a Zeiss Axio Imager M1 microscope. For further quantification of acquired images, Zen software (Carl Zeiss) was used to obtain intensities of signals and counting of positive signals. A detailed explanation on the method of quantification is described in each figure legend.

### TUNEL assay

For TUNEL staining, which was used for detecting cell death, the reaction mixture supplied by Roche’s In Situ Cell Death Detection Kit, AP (Cat. No. 11 684 809 910) was used following the protocol provided by Roche. Briefly, 10-μm thin sections of cryogenic brain tissue which were previously lightly fixed in acetone for 5 min and stored at −80 °C were thawed and fixed with 4 % paraformaldehyde for 20 to 30 min. Then, sections were permeabilized using 0.1 % Triton X-100 (Sigma-Aldrich, 9002-93-1) in sodium citrate (Fisher Scientific, 6132-04-3) with PBS for 2 min. TUNEL reaction mixture supplied by Roche’s In Situ Cell Death Detection Kit, AP was added to each section and left over night at 4 °C. Negative controls received only label solution and no terminal transferase enzyme, while positive controls were pretreated with DNase I Recombinant in 50 mM Tris-HCl and 10 mM MgCl_2_ for 10 min at room temperature to induce DNA strand breaks. TUNEL reaction mixture was then added to all samples. After washing with PBS, sections were fixed with 4 % paraformaldehyde for 10 min and then washed again with PBS. Images were captured using a Zeiss Axio Imager M1 microscope.

### Quantitative PCR

Brain and liver mRNA was extracted with TRIZOL (Invitrogen) and cDNA was synthesized using High-Capacity cDNA Reverse Transcription Kits (Applied Biosystems) according to the protocols provided by the manufacturers. Quantification of expression levels of ApoE, LRP1, TLR1, TLR2, TLR6, and of pro-inflammatory cytokines (IL-6, TNF-Α-α, IL-17, and IL-1β) was performed using specific primers and KAPA SYBR FAST qPCR Kit (KAPA biosystems) and ran on CFX96 thermocycler (Bio-Rad). Relative mRNA expression levels of genes were analyzed using the 2^^dCT^ method, normalized with GAPDH as reference gene. The fold change plotted was relative to the respective SD controls. Specificity of reaction was analyzed using melting curve analysis. Primer sequences can be viewed in supplemental Table 1.

### ELISA assay

Splenocytes were harvested from SD or HFD-fed mice after 1 year and treated with either PBS or ConA for 48–72 h. Supernatant was collected and used for ELISA analysis using the eBioscience Ready Set Go Kit. Briefly, plates were coated overnight at 4 °C with capture antibodies against IL-6, TNF-α, or IL-17 and then washed. Wells were incubated with standards and samples, washed, and then subsequently incubated with biotin conjugated detection antibodies. Plate was washed and developed with 1× TMB substrate solution (eBioscience) and the optical density (OD) was read at 450 nm using a Biotek fluorometer (Biotek). OD values were converted into absolute concentration using the standard curve.

### Detailed quantification method of immunofluorescence images

For immunofluorescence images, different methods were used for its quantitative analysis. For plaque quantification, either sizes or numbers of Thioflavin S^+^ plaques from different areas of cortex of animals of different groups were measured (area size: 6 × 10^5^ μm^2^/field, ten fields/section, blindly chosen, five animals/group). Only plaques larger than 2 × 10^3^ μm^2^ were measured and smaller plaques were not considered. For cortical thickness quantification, lengths of layer I to VI of cortex were measured from blindly chosen ten different regions of whole cortex from animals of different groups were measured for analysis (two animals/group, blindly chosen). For analysis of fragmented vessels, CD31 (vascular marker)-positive cells that lost normal brain vascular integrity (linear and smooth outlining of vessel) were considered as fragmented vessels, and the numbers were counted from cortex of animals of different groups (area size: 6 × 10^5^ μm^2^/field, ten fields/section, blindly chosen, two animals/group). All analyses (measurement of length, area size) were performed using automatic calibration function of Zen software (Carl Zeiss).

### Western blotting

Brains from WT or APP-Tg mice fed with SD or HFD were homogenized and lysed with lysis buffer containing protease inhibitor cocktail. Samples were loaded onto a 10 % SDS acrylamide gel and separated for 1 h and transferred to a nitrocellulose paper. The membrane was blocked with 1 % BSA/TBST and incubated with anti-phospho-Tau antibody (S396 from abcam, AT8 from Millipore) or anti-Tau antibody (Cell signaling) overnight at 4 °C and washed three times with TBST. For loading control, anti-GAPDH (Cell signaling) was used. Subsequently, the blot was incubated with HRP conjugated secondary antibody for an hour at room temperature and washed three times with TBST. The blot was developed with Super Signal West Pico ECL solution (Thermo scientific) and exposed to X-ray film. The film was scanned, and the intensity of each band was analyzed with Image J software.

### Statistical analyses

Data were analyzed by one-way or two-way ANOVA, followed by Bonferroni’s multiple comparison test or Student’d *t* test (two-tailed, unpaired) using GraphPad Prism 5 software (GraphPad, La Jolla, CA). Plotted data shown represents Mean ± SEM where significance is indicated by *p < 0.05, **p < 0.01, ***p < 0.001.

## Results

### Acute stage NAFLD accelerated β-amyloid plaque formation in APP-Tg mice but not in WT mice

**Fig. 1:**
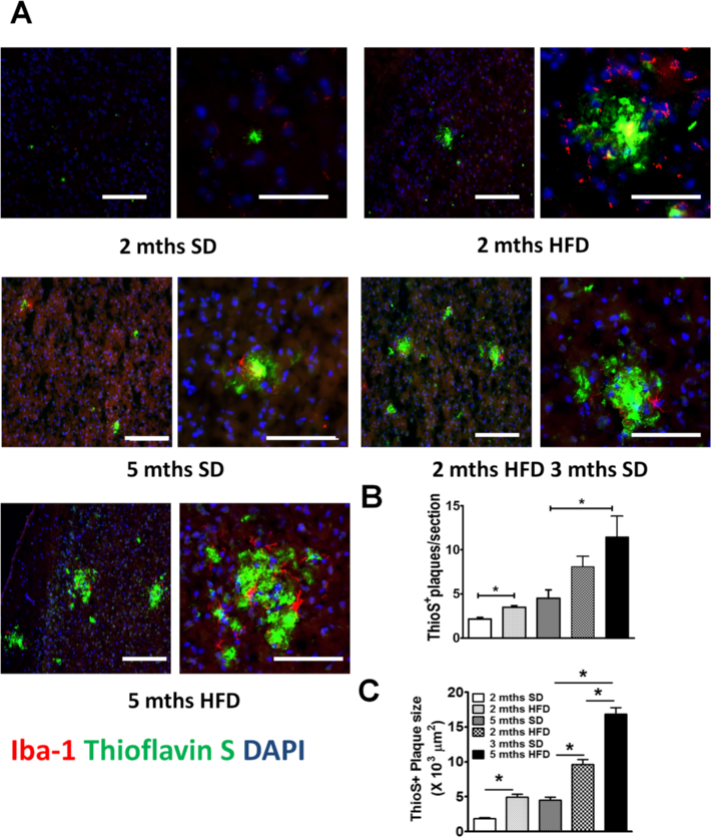
HFD accelerated β amyloid plaque burden in APP-Tg mice. **a** Representative images of Thioflavin S (*green*) and Iba-1 (*red*) stained cortical brain sections from age-matched APP-Tg mice. Nucleus was counter-stained with DAPI (*blue*). Mice were fed with SD (*left top panel*) or HFD (*right top panel*) for 2 months. A different set of mice was fed SD for five months (*left middle panel*) or HFD for 2 months followed by SD for 3 months (*right middle panel*). A different set of mice was kept on HFD for 5 months (*left bottom panel*). Images on the right side are close ups of the plaque clusters seen on the images on the *left*. Scale bar = 100 μm. **b, c** Quantification of Thioflavin S positive plaque number (**b**) and size (**c**) in cortical sections of multiple fields (ten fields/mouse) from five mice/group in one representative experiment out of four independent experiments. * indicates *p* < 0.05 (One-way ANOVA with Bonferroni post-test)

It is becoming more and more evident that diets high in fats/lipids can cause metabolic diseases such as NAFLD. Metabolic diseases are emerging as significant contributors to dementia and cognitive decline [33, 34]. To investigate the impact of acute NAFLD in AD pathogenesis, we induced NAFLD in age- and gender-matched WT and APP-Tg mice by feeding them a high-fat diet (consisting of 1 % cholesterol and 18 % triglycerides) (Table 1) beginning at 2 months of age. Mice were fed HFD for 2, 5 months, or fed HFD for 2 months and put back on standard diet (SD) for 3 months. Control mice (WT and APP-Tg mice) were fed SD for either 2 or 5 months. To determine whether HFD increased β-amyloid (Aβ) plaque burden in APP-Tg mice, or induced Aβ plaques in WT mice, we analyzed brain sections of WT and APP-Tg mice on HFD for 2 and 5 months, and of APP-Tg mice that were removed from HFD after 2 months. We observed a significant increase in Aβ plaque number in APP-Tg mice fed HFD at 2 and 5 months compared to SD-fed controls as visualized by Thioflavine S-stained brain sections (Fig. 1a, b). APP-Tg mice that were removed from HFD after 2 months had lower plaque burden than mice that were on HFD continuously for 5 months (Fig. 1a, b). Not only was the plaque burden in APP-Tg mice fed HFD for 5 months greater, the plaque sizes were larger in diameter compared to the average plaque size in control mice on SD and were inundated with activated microglial cells (Fig. 1a, c). We did not observe Aβ plaques in WT mice on HFD either at 2 or 5 months (Additional file 1: Figure S1). We conclude that HFD accelerated Aβ plaque formation in APP-Tg mice but did not induce plaques in WT controls. Moreover, removal of APP-Tg mice from HFD to SD lessened plaque load compared to mice on HFD for 5 months. This suggests that if dietary intake is corrected early in disease (prior to signs of advanced AD), signs of AD can be reversed.

**Table 1:** Fat composition of standard diet (SD) and high-fat diet (HFD)

Fat composition (%)	SD	HFD
Total fat	5.8	15.8
Cholesterol	0	1.0
Cholic acid	0	0
Triglyceride	0	18

### Both WT and APP-Tg mice were susceptible to HFD-induced NAFLD steatohepatitis and systemic inflammation

**Fig. 2:**
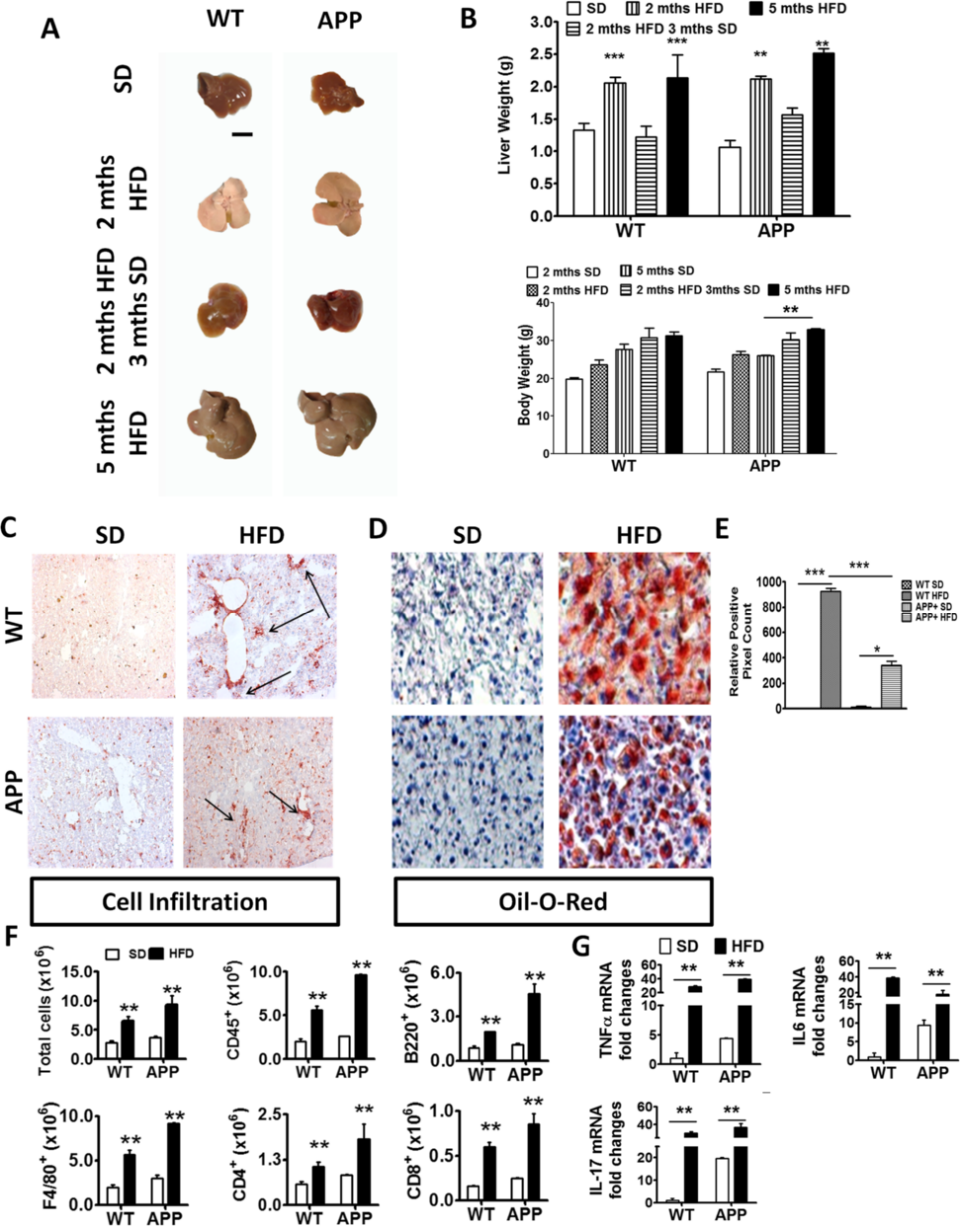
HFD induced acute liver pathology and inflammation in WT and APP-Tg and mice. **a** Representative images of livers from WT or APP-Tg mice fed with HFD or SD, with corresponding liver and body weights of their respective age-matched SD-fed controls. Scale bar indicates 5 mm (**b**). ** and *** indicate *p* < 0.01 and *p* < 0.001, respectively (two-way ANOVA with Bonferroni post-test). **c** Representative CD45^+^ staining of liver sections from WT or APP-Tg mice fed with SD or HFD for 2 months. **d** Representative oil O-red staining on frozen tissue sections of livers from WT or APP-Tg mice fed with SD or HFD for 2 months. **e** Relative positive pixel count of oil O-red staining quantified using Zen software (Carl Zeiss). * and *** indicate *p* < 0.05 and *p* < 0.001, respectively (two-way ANOVA with Bonferroni post-test). **f** Quantitative analysis of total leukocyte numbers (CD45^+^) isolated from the livers of 2-month SD or HFD-fed WT and APP-Tg mice by flow cytometry. ** indicates *p* < 0.01 (two-way ANOVA with Bonferroni post-test, *n* = 3/group). **g** TNF-α, IL-6, and IL-17 mRNA expression in livers of WT and APP-Tg mice fed with SD or HFD for 2 months. ** indicates *p* < 0.01 (two-way ANOVA with Bonferroni post-test, *n* = 3/group, representative experiment of four independent experiments)

NAFLD induces severe liver inflammation and causes significant liver damage [18]. To confirm NAFLD induction, we examined the livers of mice and observed that both WT and APP-Tg mice on HFD exhibited significant liver abnormality, characterized by severe hepatomegaly, fat accumulation, and significant increase in liver size and weight (Fig. 2a, b). Interestingly, when both WT and APP-Tg mice were removed from HFD after 2 months and put back on normal chow for 3 months, the fatty liver resolved and the size of their liver was similar to control mice that were continuously on SD (Fig. 2a, b). Notably, HFD consumption did not lead to abnormal body weight gain in WT mice. However, in APP-Tg mice, we observed statistically significant higher body weight in HFD-fed mice compared to their SD controls at 5 months (Fig. 2b). It was previously reported that HFD treatment can increase weight gain in APP-Tg mice [35].

NAFLD begins with accumulation of fat vesicles in the liver cells (steatosis) which does not disturb normal functions of the liver. However, further damage including oxidative stress and mitochondrial abnormalities can induce secondary inflammation and immune cell infiltration in the liver which is known as steatohepatitis [12, 20]. To further characterize the liver pathology in HFD-fed mice, we performed immunohistochemistry to examine immune cell infiltration in the liver (hepatitis), as well as oil red-O staining to evaluate fat deposition in the liver cells (steatosis) (Fig. 2c, d). We observed multifocal hepatitis and substantial steatosis in WT and APP-Tg mice fed with HFD, whereas the livers of mice fed with SD were devoid of hepatitis or steatosis (Fig. 2c). Although the livers of both WT and APP-Tg mice fed with HFD showed marked lipid droplet deposition, WT mice exhibited larger pockets of fat deposits than APP-Tg mice (Fig. 2d, e). Analysis of inflammatory leukocyte subpopulations revealed an increase in CD4^+^, CD8^+^, B220^+^, and F4/80^+^ cells in the livers of both WT and APP-Tg mice fed with HFD compared to their respective SD-fed controls (Fig. 2f). We next determined whether NAFLD induced a systemic pro-inflammatory state in HFD mice. We performed gene expression analysis of pro-inflammatory cytokines from the livers of WT and App-Tg mice fed with SD or HFD using quantitative PCR (qPCR). We observed increased expression of TNF-α, IL-6 and IL-17 in both WT and APP-Tg mice on HFD compared to SD controls (Fig. 2g). These findings indicate that HFD-induced NAFLD caused an acute inflammatory state, in the absence of increased weight gain, which accelerated plaque formation in APP-Tg mice but not in WT mice.

### HFD induced neuro-inflammation in both WT and APP-Tg mice in acute stage AFLD

**Fig. 3:**
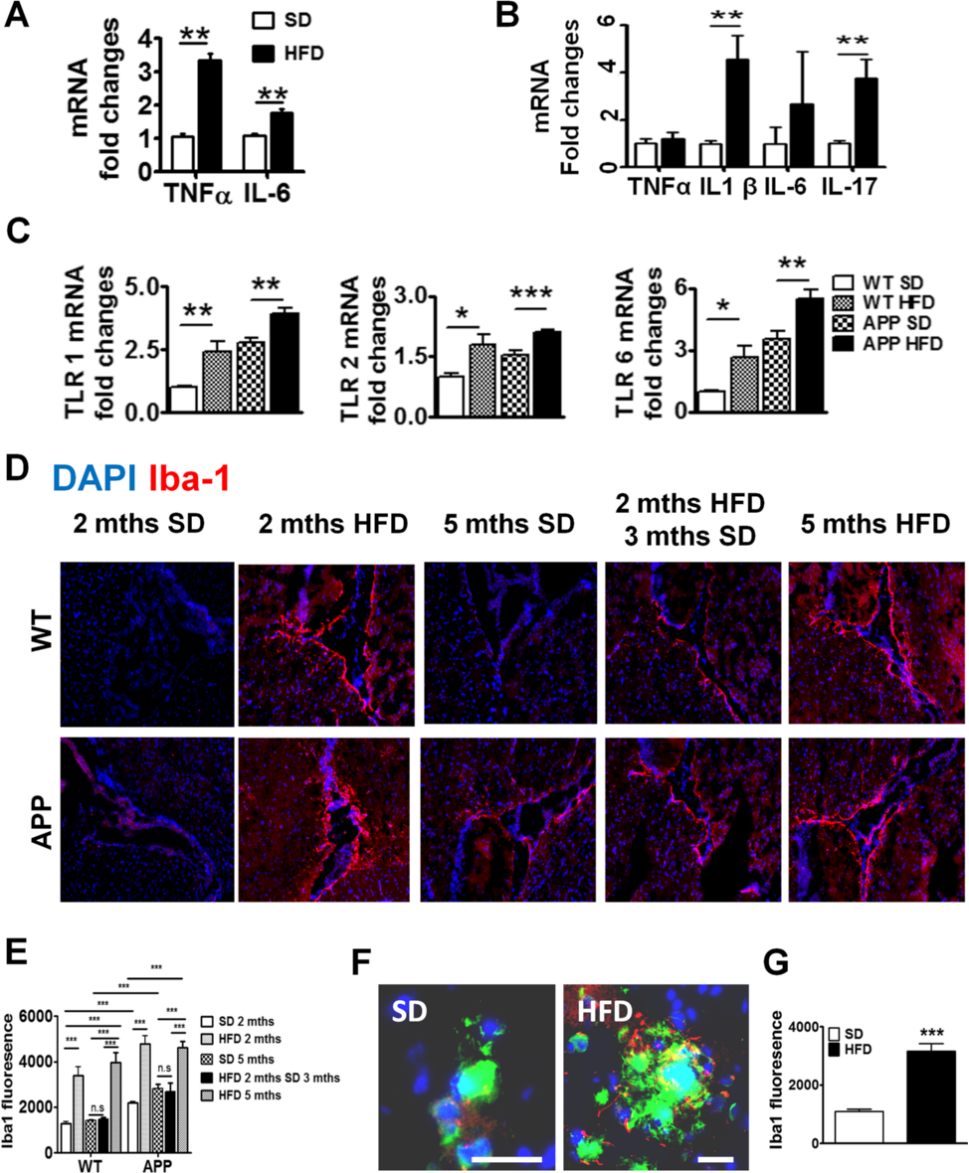
Brains of HFD-fed mice have increased inflammation and microglial activation. mRNA expression of pro-inflammatory cytokine in brains of APP-Tg (**a**) and WT mice (**b**). ** indicates *p* < 0.01 (two-tailed student *t* test, *n* = 2 performed in triplicates representative of four independent experiments). **c** Brain mRNA expression of TLR1, TLR2, and TLR6 of WT or APP-Tg mice after being on SD or HFD for 2 months. *, **, *** indicate *p* < 0.05, *p* < 0.01, and *p* < 0.001 (two-tailed student *t* test, *n* = 2 performed in triplicates representative of four independent experiments). **d** Representative images of Iba-1 (*red*) near the choroid plexus from WT mice and APP-Tg mice fed with SD or HFD for 2 or 5 months, or initially with HFD for 2 months and then put back on SD for 3 months. Nucleus was counter-stained with DAPI (*blue*). **e** Intensity of Iba-1 expression from **d** measured from different fields (*n* = 20, three mice/group) using Zen Software (Carl Zeiss). ** and *** indicate where *p* < 0.01 and *p* < 0.001, respectively. (two-way ANOVA with Bonferroni post-test) **f** Representative image of relative localization of Iba-1 (*red*) with β- amyloid plaque (*green*) from cortices of brains of mice fed with SD or HFD for 2 months. Nucleus was counter-stained with DAPI (*blue*). Scale bar = 50 μm. **g** Intensity of Iba-1 signal from (**f**) was measured from different plaques using Zen Software (Carl Zeiss) (*n* = 20, five mice/group). *** indicates *p* < 0.001 (two-tailed student’s *t* test)

Peripheral inflammation, that is, inflammation induced outside the CNS, has long been associated with inducing CNS inflammation leading to neurodegeneration [12, 36]. To determine whether NAFLD-induced inflammation (which initially starts in the liver) induces CNS inflammation in mice fed with HFD that may account for the accelerated plaque burden in APP-Tg mice (Fig. 1), we performed cytokine gene expression analysis on the brains of WT and APP-Tg mice fed with either HFD or SD. We observed higher levels of TNF-α and IL-6 mRNA in the brains of APP-Tg mice on HFD compared to SD controls (Fig. 3a). Interestingly, WT mice on HFD expressed higher levels of IL-1β and IL-17 mRNA in the CNS compared to SD controls (Fig. 3b). This indicates that peripheral inflammation has a significant effect on induction of CNS inflammation (Fig. 3b).

Toll-like receptors (TLRs), which are innate immune receptors that recognize microbial components called pathogen-associated molecular patterns (PAMPS) or endogenous ligands released by necrotic or injured cells called damage-associated molecular patterns (DAMPS), have been recently described in CNS inflammation [37]. TLR association with specific DAMPs leads to receptor activation, which subsequently leads to initiation of an inflammatory cascade such as the one observed in Fig. 3a, b. We investigated whether TLRs were altered in the CNS of WT or APP-Tg mice fed with HFD compared to controls during acute NAFLD. We focused on TLRs 1, 2, and 6 as these TLRs are known to recognize lipoproteins and glycolipids among others [38]. We observed significant upregulation of TLR 1, 2, and 6 in the brains of WT and APP-Tg mice fed with HFD compared to SD controls (Fig. 3c). This confirms that HFD induced a potent inflammatory cascade inducing TLRs and culminating in secretion of pro-inflammatory mediators in the CNS.

Microgliosis is characterized by an increase in the number activated microglial cells accumulating around CNS lesions or during CNS injury and is believed to contribute to CNS pathology in models of neuro-inflammation [39, 40]. We found that WT and APP-Tg mice fed with HFD showed markedly pronounced staining for the activated microglial marker Iba-1, in areas surrounding the choroid plexus (Fig. 3d and Additional file 2: Figure S2). Interestingly, despite the absence of plaques in WT mice on HFD, these mice showed intense Iba-1 staining and increased microglial cell numbers, suggesting that the CNS of these mice is being primed for a neuroinflammatory or pathological event (Fig. 3d, e). Interestingly, Iba-1 staining was significantly reduced in mice that were removed from HFD after 2 months, indicating that HFD-induced systemic inflammation is the major factor contributing to CNS inflammation and microglial activation (Fig. 3d, e). We also observed Iba-1 staining in APP-Tg mice fed with SD, albeit to a lesser extent (Fig. 3d, e), suggesting pre-existing pro-inflammatory conditions in the CNS of these mice as previously reported [41]. Since microglial cell activation is higher in the choroid plexus of HFD-fed mice, we asked whether such increased microglial activation is observed in plaque-rich areas in the brains of APP-Tg mice fed with HFD. Indeed, we observed higher microglial activation in plaque-rich regions in brains from mice fed HFD for 2 months compared to those that were fed SD (Fig. 3f, g). Increased microglial activation was also observed in cortical and hippocampal areas devoid of β-amyloid plaques in HFD-fed WT and APP-Tg mice compared to their SD-fed controls (Additional file 3: Figure S3 A–D). This indicates that HFD-induced NAFLD can trigger generalized microglial activation in the brains of both WT and APP-Tg mice.

### Impact of HFD on chronic disease: NAFLD caused advanced signs of AD in both WT and APP-Tg mice 1 year later

**Fig. 4:**
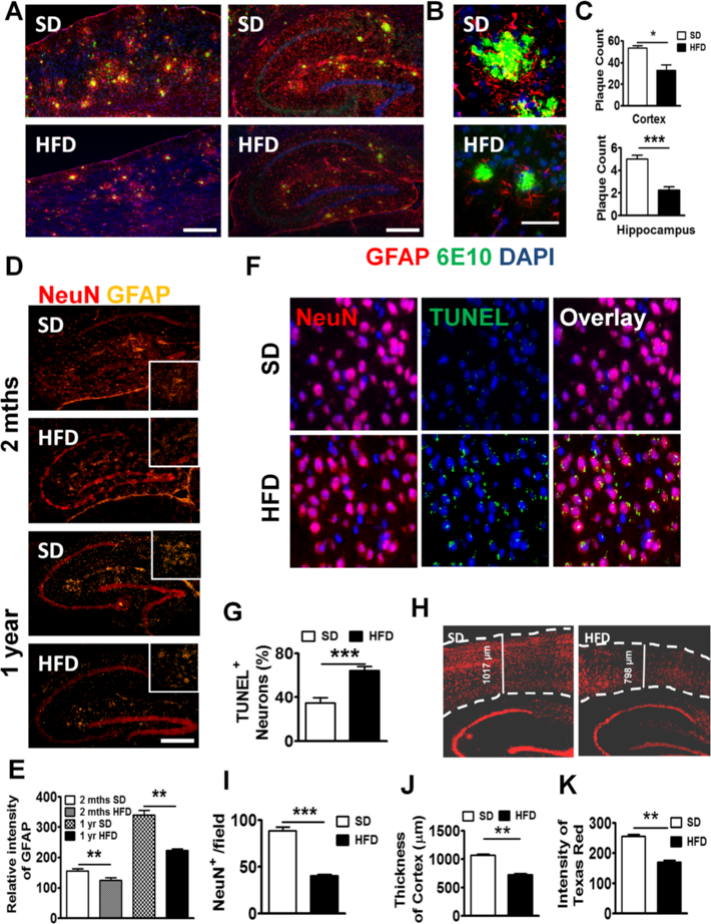
Long-term HFD decreased NeuN^+^ cells, Aβ plaques, and astrocytes in APP-Tg mice. **a** Representative images of cortex (*left panel*) or hippocampus (*right panel*) from APP-Tg mice fed with SD or HFD for 1 year, stained with anti-6E10 anti-β-amyloid antibody (*green*), anti-GFAP (*red*), and DAPI (*blue*). Scale bar = 200 μm. **b** Representative zoomed-in images of reactive astrocyte (*red*) around plaque (*green*) from the cortex of SD or HFD-fed APP-Tg mice. Scale bar = 50 μm. **c** Number of plaques from different regions of cortex (*n* = 10) or hippocampus (*n* = 10). * indicates *p* < 0.05 (two-tailed student’s *t* test, *n* = 2 mice/group, representative of two independent experiments). **d** Representative with SD or HFD for 2 months (*top panel*) or 1 year (*bottom panel*) stained with anti-NeuN (*red*), and anti-GFAP antibody (*gold*). Zoomed-in image was inserted as inlet. **e** Relative intensity of GFAP signal from **d** (*n* = 10). ** indicates *p* < 0.01 (two-tailed student’s *t* test, twice mice/group, representative of two independent experiments). Representative images (**f**) and quantitative analysis (**g**) of images from brain cortex of APP-Tg mice fed with SD or HFD stained with NeuN (*red*), or TUNEL (*green*). TUNEL/NeuN double positive cells were counted from ten different fields and quantified as a percentage of total cell count. *** indicates *p* < 0.001 (two-tailed student’s *t* test, two mice/group). **h** Representative image of brain sections from SD or HFD-fed APP-Tg mice stained with anti-NeuN antibody (*red*). *Dotted lines* indicate the outline of NeuN-positive signal in cerebral cortex. **i** Quantitative analysis of **h** *** indicates *p* < 0.001 (two-tailed student’s *t* test, two mice/group). **j** Cortical thickness of brains from SD or HFD-fed mice measured from ten different fields. ** indicates *p* < 0.001 (two-tailed student’s *t* test, two mice/group). **k** Quantitative analysis of **h**, showing intensity of NeuN-positive signal measured from ten different fields of dentate gyrus from mice fed SD or HFD. ** indicates *p* < 0.001 (two-tailed student’s *t* test, two mice/group)

To mimic the life-long diet pattern in humans, we kept WT and APP-Tg mice on HFD for up to 1 year to evaluate its impact on systemic as well as on brain inflammation and subsequent AD pathogenesis. First, we analyzed the accumulation of fat deposition (steatosis) in the liver after 1 year of HFD treatment in both WT and APP-Tg mice. We observed significantly higher accumulation of fat in both WT and APP-Tg mice fed with HFD, indicating ongoing NAFLD (Additional file 4: Figure S4). Because we observed accelerated plaque formation in APP-Tg mice at 2 and 5 months on HFD that is comparable with the plaque loads and sizes seen in these mice around 18–24 months on SD, we anticipated an even greater plaque burden in APP-Tg mice fed with HFD for 1 year. Surprisingly, instead of increased plaques, we observed a significant decrease in overall plaque load in APP-Tg mice on HFD compared to those on SD (Fig. 4a). We examined and quantified Aβ plaque deposition in the cortex and hippocampus (Fig. 4a). We observed a dramatic reduction in Aβ plaques in HFD-fed mice compared to SD-fed controls in these regions (Fig. 4a, c). Also, close-up images of plaques from these mice showed increased reactive astrocyte infiltration in SD-fed mice compared to HFD-fed mice (Fig. 4b). Moreover, from 2 months (Fig. 4d upper panel) and up to 1 year (Fig. 4d bottom panel) of HFD treatment, we observed a reduction of GFAP signal in the hippocampus compared to control treatment (Fig. 4e). We hypothesized that the reduced plaque load may be a result of neuronal and/or glial cell death or due to increased plaque clearance by astrocytes, which can exert a protective response to fibrillar Aβ by removing it from the CNS [42]. To determine whether the decreased plaque load in the CNS of APP-Tg mice was the result of neuronal death leading to a decrease in Aβ production, we stained frozen brain sections with antibodies to TUNEL which stains DNA in dead or dying cells, and to NeuN which stains neurons. We observed higher incidence of TUNEL-positive NeuN cells in HFD-fed mice compared to SD-fed APP-Tg mice (Fig. 4f, g). Further, quantification of neuronal cells in the cortex showed that they were significantly decreased in APP-Tg mice fed with HFD compared to SD controls (Fig. 4h, i). Also, the thickness of the cortex was shrunken in APP-Tg mice fed with HFD compared to SD controls (Fig. 4h, j). Moreover, the intensity of NeuN-positive signal in the dentate gyrus was reduced in APP-Tg mice fed with HFD compared to SD-fed controls (Fig. 4h, k). Since astrocytes and neurons are major producers of Aβ, these findings suggest that reduction in neuronal cells and astrocytes may be responsible for the reduced plaque burden.

### Long-term HFD-induced AD plaques and neuronal cell loss in WT mice

**Fig. 5:**
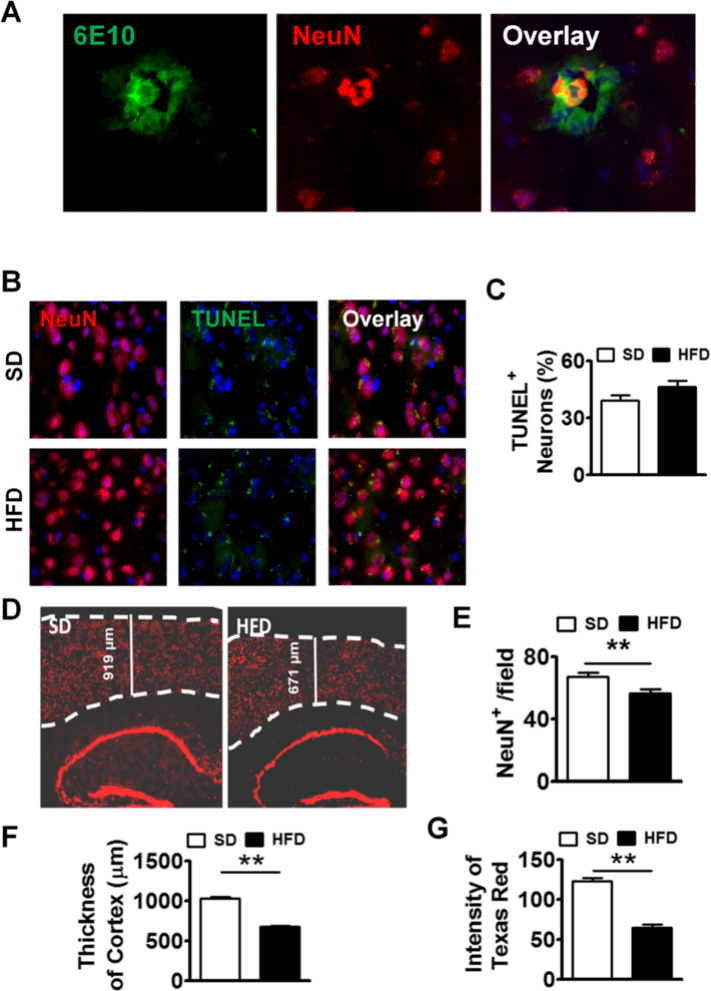
Long-term HFD induced Aβ plaque formation in brains of WT mice. **a** Representative images of brain sections from HFD-fed WT mice stained with anti-NeuN (*red*) and anti-6E10 (*green*). **b** Representative images of brain sections (cortex) from SD or HFD-fed WT mice double-stained with anti-NeuN (*red*) and TUNEL (*green*). **c** TUNEL/NeuN double positive cells were counted from ten different fields and quantified as a percentage of total cell count. **d** Representative image of brain sections from SD or HFD-fed WT mice stained with anti-NeuN antibody (*dotted lines* indicate the outline of NeuN-positive signal in the cerebral cortex. **e** Quantitative analysis of **d**, showing numbers of NeuN^+^ cells from ten fields of the brain. ** indicates *p* < 0.01 (two-tailed student’s *t* test, two mice/group). **f** Comparison of cortical thickness of brains from SD or HFD-fed mice. Thickness of cortex was measured from ten different fields. ** indicates *p* < 0.01 (two-tailed student’s *t* test, two mice/group). **g** Quantitative analysis of **d**, capturing the intensity of NeuN-positive signal measured from ten different fields of the dentate gyrus from brains of mice fed with SD or HFD. ** indicates *p* < 0.01 (two-tailed student’s *t* test, two mice/group). These are representative findings from two independent experiments

WT mice developed inflammation both in the periphery and in the CNS similar to and to some degree greater than APP-Tg mice in acute NAFLD (Figs. 2 and 3). Brains of WT mice had increased numbers of activated microglial cells and high levels of pro-inflammatory mediators but no evidence of plaque induction during acute NAFLD. We examined brains of WT mice to determine whether aging (6 months later) induced Aβ plaques in the presence of HFD. Indeed, we observed plaque formation in the cerebral cortex of WT mice fed with HFD (Fig. 5a). This finding is significant because it indicates that HFD-induced chronic inflammation plus aging is sufficient to induce signs of AD in mice lacking genetic predisposition to AD (Fig. 5a). To test whether WT mice also show increased neuronal apoptosis as was observed in APP-Tg mice, we performed TUNEL/NeuN double-staining. Similar to APP-Tg mice, we observed a higher incidence of TUNEL-positive NeuN staining (not significant) in WT mice on HFD compared to SD (Fig. 5b, c). To test if HFD induced neuronal loss, we quantified the number of NeuN-positive cells in different brain regions. Indeed, we observed a statistically significant reduction of NeuN-positive signal in the cortex (Fig. 5d, e) and reduced cortical thickness (Fig. 5d, f) in WT mice fed with HFD compared to SD controls. Also, the intensity of NeuN-positive signal in the dentate gyrus was reduced in WT mice fed with HFD compared to SD controls (Fig. 5d, g). This suggests that long-term HFD treatment in WT mice is sufficient to induce plaque formation and neuronal loss. Interestingly, we did not observe any decrease or reduction in astrocytes or Aβ plaque deposition in the brains of WT mice on SD vs. HFD as was observed in APP-Tg mice (Additional file 5: Figure S5).

### Long-term HFD induced advanced signs of AD in WT and APP-Tg mice

**Fig. 6:**
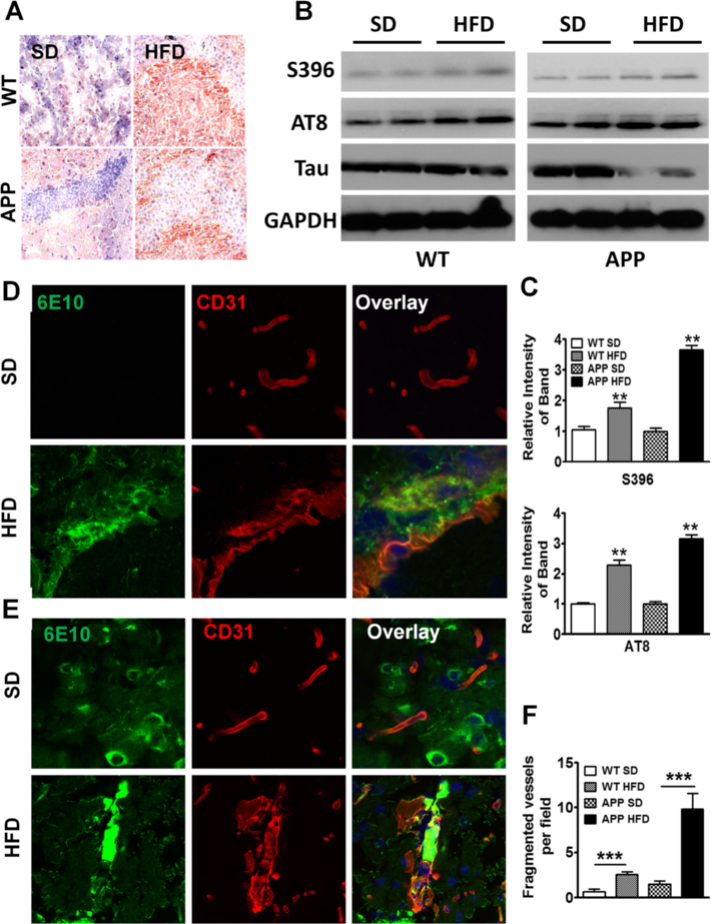
Long-term HFD induced advanced signs of AD in WT and APP-Tg mice. **a** Representative images of hippocampus from WT and APP-Tg mice fed with SD or HFD for 1 year, stained with anti-phosphorylated-Tau (S396) antibody. **b, c** Western blot analysis of brain homogenate of WT or APP-Tg mice fed with SD or HFD for 1 year (**b**). Phosphorylated-Tau was detected with S396 or AT8 antibody and total Tau protein was detected with anti-Tau antibody. GAPDH was used as loading control. Intensity of phospho-Tau was normalized by that of Tau protein which was further normalized by intensity of GAPDH and depicted in graph (**c**). ** indicates *p* < 0.01. (two-tailed student’s *t* test, *n* = 2). **d** Representative brain sections from WT mice fed SD or HFD and stained with anti-CD31 (*red*) and anti-β- amyloid antibody, 6E10 (*green*) to assess the degree of cerebral amyloid angiopathy (CAA) and vasculopathy. The *top* three panels depict SD controls, the *bottom* three panels show HFD-induced CAA. **e** Representative brain images from APP-Tg mice double-stained with antibodies to β-amyloid (6E10) and CD31 (vasculature) for SD (*top* three panels) or HFD (*bottom* three panels) to evaluate CAA. **f** Quantification of fragmented vessels taken over multiple microscopic fields throughout the brains of WT and APP-Tg mice fed with SD or HFD. *** indicates *p* < 0.001 (Two-tailed student’s *t* test, ten fields/mouse, *n* = 2 mice/group)

In addition to Aβ senile plaque deposits, aggregation of neurofibrillary tangles composed of hyperphosphorylated-Tau (pTau) is a prominent hallmark of advanced AD. Tau is the microtubule-associated protein (MAP) that is involved in the delivery of neurotransmitter signals through microtubule tracks in axons [43, 44]. In its hyperphosphorylated state such as in AD, or tauopathies, Tau is aggregated and unable to relay proper neurotransmission, leading to neuronal dysfunction [10, 11, 45]. To test whether tauopathy is induced in APP-Tg mice 1 year after being on HFD, we stained the brains of mice fed with either HFD or SD with an antibody that binds to pTau. We observed very intense pTau staining in the hippocampus and cortex of APP-Tg mice but not in SD controls, indicating that long-term HFD induced major advanced signs of neurodegeneration (Fig. 6a). We next determined whether WT mice on HFD also exhibit pTau staining. Indeed, similar to APP-Tg mice, WT mice exhibited intense pTau staining in the dentate gyrus that was not observed in SD-fed mice (Fig. 6a). This was further confirmed by Western blot using pTau-specific antibodies (S396 and AT8) showing increased ratio of pTau/Tau in both HFD-fed WT and APP-Tg mice compared to their SD controls (Fig. 6b, c). These findings strongly indicate that HFD induced major advanced signs of neurodegeneration, not only in animals predisposed to AD (APP-Tg mice) but was sufficient to induce them in WT mice as they age.

We next investigated whether cerebral amyloid angiopathy (CAA), a sign of advanced AD characterized by accumulation of Aβ_1–40_ in the brain vasculature and believed to cause micro-infarction leading to neurological dysfunction and neuronal death, was induced by HFD [46, 47]. Aβ deposition in cerebral vessels lowers the force of Aβ clearance, resulting in increased parenchymal Aβ build-up. We examined brains of mice after double-staining with antibodies to Aβ and CD31 (marker for the endothelial vasculature). We observed intense and frequent signs of CAA, evidenced by Aβ accumulation in the vessels of WT and APP-Tg mice fed with HFD but not in SD controls (Fig. 6d, e). Throughout the brains of WT and APP-Tg mice, we observed what appears to be a high frequency of broken or fragmented vessels (Fig. 6f). These findings indicate that HFD can induce signs of advanced AD in WT mice and profoundly accelerate signs of advanced AD in APP-Tg mice fed with HFD.

### HFD decreased LRP1 and increased the inflammatory profile in the brains of WT and APP-Tg mice during chronic NAFLD

**Fig. 7:**
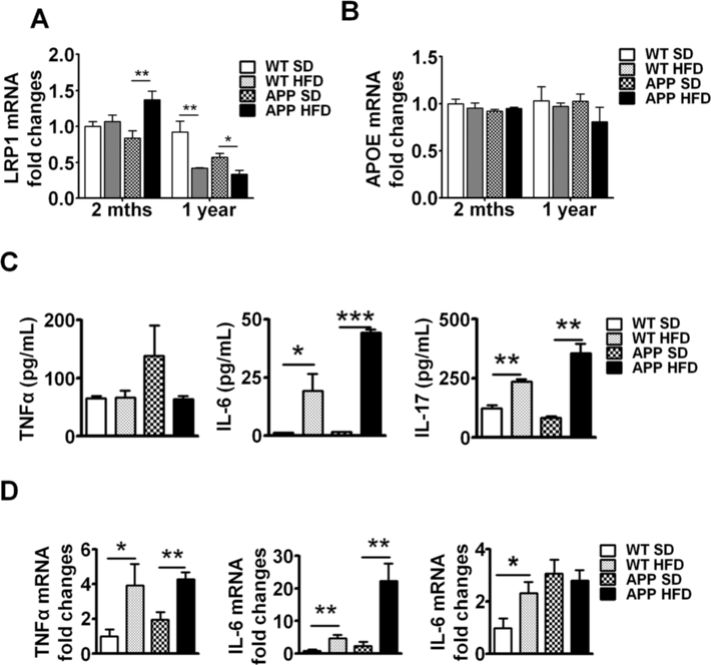
Long-term HFD decreased LRP1 and maintained chronic inflammation in brains of WT and APP-Tg mice mRNA expression of LRP1 (**a**) or ApoE (**b**) was analyzed in the brains of mice fed SD or HFD for 2 months or 1 year by quantitative real-time PCR. * and ** indicate *p* < 0.05 and *p* < 0.01, respectively (two-tailed student’s *t* test, *n* = 2 mice/group performed in triplicates, representative of two independent experiments). **c** Splenocytes from mice fed SD or HFD for 1 year were stimulated with PMA-ionomycin for 3 days, supernatants were collected and pro-inflammatory cytokines (TNF-α, IL-6, IL-17) were quantified by ELISA. *, **, and *** indicate *p* < 0.05, *p* < 0.01, and *p* < 0.001, respectively (two-tailed student’s *t* test, *n* = 2 mice/group performed in triplicates, representative of two independent experiments). **d** mRNA expression level of pro-inflammatory cytokines (TNF-α, IL-6, IL17) was analyzed in brain samples from mice fed HFD or SD for 1 year by quantitative real-time PCR * and ** indicate *p* < 0.05 and *p* < 0.01, respectively (two-tailed student’s *t* test, *n* = 2 mice/group performed in triplicates, representative of two independent experiments)

LRP1 is highly expressed in, neurons and glial cells, as well as in the liver and vascular smooth muscle, and functions in the clearance and trafficking of Aβ from the CNS. However, studies show that LRP1 is also involved in Aβ production [31]. Thus, LRP1 involvement in Aβ synthesis and clearance makes it a prime target in AD pathogenesis. Deletion of LRP1 exacerbated Aβ deposition and increased CAA (Kanekiyo et al., 2012). We investigated whether LRP1 expression is altered in the CNS of HFD-fed mice both at early (acute NAFLD, 2 months) and chronic NAFLD (after 1 year on HFD) compared to SD controls. We observed increased LRP1 expression in the brains of APP-Tg mice fed with HFD but not in HFD WT mice (Fig. 7a). However, in chronic NAFLD, both WT and APP-Tg mice fed with HFD expressed significantly lower levels of LRP1 in CNS tissue compared to SD controls (Fig. 7a). This decrease in LRP1 expression is consistent with the increased neurodegenerative changes observed in chronic disease and may reflect a protective role for LRP1 in AD [31].

ApoE is a known risk factor for AD. Its variant ApoE4 increases the risk of AD and accelerates AD onset (Bu, 2009; Liu et al., 2013). ApoE is a ligand for LRP1, and its main function is the cellular transfer of lipids through LRP1 on the cell surface. There is mixed evidence as to whether ApoE is involved in binding and/or clearance of Aβ [48]. It is speculated that ApoE may inhibit or facilitate LRP1-mediated endocytosis of Aβ [49]. We therefore analyzed ApoE expression in both acute and chronic NAFLD and found no difference in ApoE expression in acute or chronic NAFLD in WT or APP-Tg mice (Fig. 7b). This indicates that ApoE expression/function remains relatively unchanged despite accelerated signs of neurodegeneration and neuronal loss. In contrast, LRP1 expression increased in acute/early phase of NAFLD in APP-Tg mice, but was unchanged in WT mice on HFD. LRP1 levels decreased significantly during chronic NAFLD in both WT and APP-Tg mice on HFD. This leaves us to speculate that a decrease in LRP1 expression/function is linked to advanced signs of AD.

To determine whether systemic inflammation was maintained through the chronic phase of NAFLD, we examined the cytokine profiles of lymphocytes in peripheral lymphoid organs of APP-Tg mice after 1 year on SD or HFD. We observed increased TNF-α, IL-6, and IL-17 in APP-Tg mice fed with HFD compared to SD controls (Fig. 7c). Similarly, lymphocytes from WT mice fed with HFD secreted high levels of the pro-inflammatory cytokines TNF-α, IL-6, and IL-17 compared to their SD counterparts (Fig. 7c). To determine whether this chronic HFD-induced systemic inflammatory profile is consistent with the inflammatory profile of the CNS, we performed quantitative RT-PCR of these cytokines on brain samples. Indeed, we observed that these cytokines were highly upregulated in the brains of WT and APP-Tg mice fed with HFD compared to their SD counterparts (Fig. 7d). Together, these results suggest that augmented systemic immune responses induced by HFD have a sustained impact on increased brain inflammatory response and profoundly impact neurodegeneration (Fig. 7d).

## Discussion

We demonstrate that a modest increase in dietary lipids fed to WT or APP-Tg mice induced acute inflammation of the liver followed by a chronic inflammatory state. This HFD-induced acute inflammation was characterized by invasion of inflammatory immune cells into the liver parenchyma and increased production of pro-inflammatory cytokines by immune cells locally (liver) and systemically (lymphoid organs). Concomitant with this increased peripheral inflammatory state, we observed profound acceleration in neurodegenerative signs and increased neuro-inflammation in APP-Tg mice compared to mice that remained on SD. We observed acceleration in β-amyloid plaque formation leading to higher plaque loads, larger plaque size, and increased microgliosis, and astroglyosis in APP-Tg mice. In AD patients’ brain, activated microglial cells have been observed around plaques and were initially thought to be a clearance mechanism to remove Aβ plaques from the CNS. However, these activated microglial cells did not reduce plaque burden [41], but induced neuronal damage instead. In our study, APP-Tg mice on SD exhibited activated microglial cell accumulation in the brain which is consistent with a previous report of a pro-inflammatory state in these mice [42]. Compared to SD-fed mice, microglial cell accumulation was increased by about fourfold in HFD-fed mice, and this correlated with increased pro-inflammatory cytokine expression in these mice. This increase in both CNS and peripheral inflammation was reversed when mice were removed from HFD and put back on SD; we observed decreased activated microglial cell numbers, decreased liver pathology, and decreased pro-inflammatory cytokines in the CNS. This reversal in inflammation was consistent with reduced Aβ plaque burden. This indicates that induction of NAFLD in the liver may be an important factor that can induce the accelerated signs of AD observed in APP-Tg mice.

Interestingly, WT mice on HFD exhibited a similar increase in activated microglia accumulation even though they did not exhibit signs of Aβ plaque deposition at 2 or 5 months on HFD. WT mice on HFD exhibited more pronounced lipid deposits in liver cells than APP-Tg mice, but they did not develop Aβ plaques in the acute phase of NAFLD. WT mice fed with HFD showed increased pro-inflammatory cytokine gene expression (TNF-α, Il-6 and IL-17) in the liver and in peripheral lymphoid organs, and liver pathology was more pronounced in WT mice, with higher immune cell infiltration in the liver parenchyma. Both IL-6 and Il-17 pro-inflammatory cytokines were highly pronounced in WT mice but were almost absent in SD controls. Il-6 and Il-17 play pathogenic roles in multiple sclerosis [50, 51]. The profound increase in the expression of these cytokine genes strictly coincides with increased activated microglia in the CNS, potentially setting the stage for the neurodegeneration we observed in WT mice on HFD 1 year later. Moreover, when WT mice were removed from HFD diet and placed on SD, inflammation ceased, liver pathology was reverted, and activated microglial cells were absent. Of interest was the absence of plaques in these WT mice even though they showed all other signs of inflammation observed in the APP-Tg animals. This indicates that HFD-induced systemic inflammation primed the CNS for the neurodegeneration which we later observed. These studies suggest that AD can be induced and driven by acute-chronic systemic inflammation in individuals that are not otherwise genetically predisposed. It also indicates that early intervention can reverse the process.

The brains of APP-Tg mice on long-term HFD had significantly less plaques compared to SD-fed mice. APP-Tg mice showed a dramatic decrease in Aβ plaques in the hippocampus, the olfactory bulb, and the mid brain. Also striking was the absence of astrocytic tracks in brain areas where Aβ plaques were absent or diminished, such as the hippocampus and midbrain. Moreover, we observed neuronal loss and increased apoptotic neurons in hippocampus and mid brain. This was not the case for older mice (15–24 months) on SD, indicating that aging alone is not responsible for the loss of astrocytes. As discussed above, removal from HFD reduced plaque load and decreased inflammatory signals. Thus, astrocytic loss may be a result of accelerated AD pathology resulting in CNS toxicity. Astrocyte death can lead to neuronal death because of the critical role they play in neuronal function [42]. Increased neuronal death may also explain the decreased plaque load in chronic AD due to decreased APP production. Based on these studies, we conclude that chronic inflammation induced outside the CNS (in the liver) is sufficient to induce AD-like symptoms in the absence of predisposing genetic factors.

Signs of CAA and hyperphosphorylated-Tau (pTau) expression are representative of advanced AD. pTau aggregates and is unable to signal proper neurotransmission, leading to neuronal dysfunction; and CAA results in a lack of Aβ clearance from the CNS. We observed strong expression of pTau and evidence of CAA in both WT and APP-Tg mice on HFD but not in SD controls. pTau was increased in HFD-fed WT and APP-Tg mice compared to SD controls, even though Aβ was induced much later in WT mice. Thus, if CAA and tauopathy represents advanced AD, one may argue that AD accelerated more dramatically in WT mice than APP-Tg mice, and it may be regulated differently in genetically predisposed (APP-Tg) vs. non-predisposed individuals (WT). CAA involves deposition of amyloid in cerebral vasculature and is a hallmark of advanced AD resulting in pathological changes in cerebral blood vessels referred to as vasculopathies. Signs of CAA were more extensive in APP-Tg mice compared to WT mice on HFD. These findings clearly indicate that HFD-induced inflammation can result in significant CNS destruction and pathology over a lifetime.

Low-density lipoprotein receptor-1 (LRP1) is involved in a number of pathways linked to AD pathogenesis and has multiple functions. It is most highly expressed in brain, liver, and lungs. In the brain, it is highly expressed in glial cells, neurons, and cells of the cerebral vasculature. LRP1 can directly regulate gene expression through its intracellular domain and can regulate the endocytosis of many diverse ligands including ApoE, APP, and Aβ. LRP1 appears to have bimodal opposing functions linked to AD pathogenesis. It is involved in Aβ clearance and Aβ production. It mediates Aβ clearance by cellular uptake followed by lysosomal degradation and/or transcytosis of intact Aβ across the BBB to the circulation and consequent peripheral clearance [49]. We observed increased LRP1 expression in APP-Tg mice on HFD, but not in WT mice, during the acute phase of NAFLD. Increased LRP1 expression may represent its increased function in clearance of Aβ from the CNS in APP-Tg mice during accelerated Aβ production. It is possible that LRP1 did not increase in WT mice on HFD due to the absence of Aβ in the CNS of these mice during acute NAFLD. However, during chronic NAFLD, we observed a significant decrease in LRP1 in the CNS of both WT and APP-Tg mice on HFD. This decrease coincides with advanced signs of AD, including reduced Aβ plaque load, reduced number of neurons and astrocytes, and increased vascular destruction, suggesting that LRP1 plays a protective role in AD. It is possible that the reduction in LRP1 expression during chronic NAFLD may be a result of glial and neuronal cell loss, as these cells abundantly express LRP1. Alternatively, advanced AD may have rendered LRP1 defective in clearing Aβ from the CNS.

It is interesting that no change in ApoE expression was observed during acute or chronic stages of NAFLD, despite significant neuronal and glial cell loss. The major function of ApoE is to transport cholesterol and other lipids in plasma and brain through a variety of cell surface receptors including LRP1 [49]. Astrocytes are the main source of ApoE [25, 52]. Since cholesterol is a critical component of glial and neuronal cell membrane including the myelin sheath, it is possible that a reduction of GFAP^+^ astrocytes resulted in limited cholesterol production needed for membrane synthesis and CNS repair. However, this is unlikely since such a reduction may have resulted in decreased ApoE. Other likely scenarios for no change in ApoE include its function in cholesterol trafficking was unhindered, whereas its function in Aβ trafficking was defective, or that the reduced plaque burden in chronic NAFLD was the result of increased Aβ trafficking by ApoE. The involvement of Aβ, LRP1, and ApoE in neurodegeneration and AD pathogenesis is quite complex and needs further investigation beyond these studies.

Our studies address a growing problem in our society that relates to metabolic syndromes due to diets high in fat/lipid consumption and their impact on neurodegeneration. NAFLD is prevalent in as much as a third of the world’s population. Similarly, AD frequency is rapidly growing. We showed that a modest increase in dietary lipid content caused increased systemic inflammation followed by increased neuro-inflammation and accelerated AD signs in APP-Tg mice. More importantly, we showed that WT mice become susceptible to developing Aβ plaques after long-term HFD intake and developed advanced cellular signs of AD. Moreover, APP-Tg mice on HFD exhibit severe CNS damage stemming from the effects of chronic HFD. These findings highlight a growing problem in our society whereby consumption of foods high in lipids over a lifetime can have detrimental consequences such as accelerating signs of AD in potentially susceptible individuals or inducing them in those that are not susceptible. An important and critical finding of these studies is that change from HFD to SD, before irreversible CNS damage sets in, completely reverses signs of AD. This suggests that life style changes such as reducing one’s lipid/fat intake can have a profound impact on disease outcome. Recent studies from others showed that a high-cholesterol diet (5 %) fed to mice can induce advanced pathological signs of AD [53–55]. Similar to our findings, these studies showed that diets high in cholesterol increased hyperphosphorylated-Tau deposition, and decreased cognitive function in both WT and AD models [53–55]. Also, one of the studies showed increased ventricular volume which is reminiscent of the decreased cortical thickness and increased neuronal apoptosis we observed in HFD-fed WT and APP-Tg mice [55]. These findings are in line with ours and highlight a critical role for a diet high in fats and lipids in inducing pathological outcomes. However, our model differs in the cholesterol content. We fed mice a diet containing 1 % cholesterol plus 18 % triglyceride which consistently induces NAFLD that is characterized by acute inflammation and significant liver damage in mice.

## Conclusions

In summary, chronic high-fat diet induced NAFLD, and its secondary neuro-inflammation is sufficient to cause neurodegeneration in both WT and APP-tg mice. We believe this study stands to benefit millions of people who are in danger of developing AD or people currently suffering from early signs of AD or dementia. Future studies hinge on elucidating the fine changes conferred upon neuronal function resulting in their loss or dysfunction, correlating with behavioral and cognitive defects, and defining cerebral vasculopathies resulting from these events.

## Additional files

Additional file 1: Table S1. Sequences of primers used for quantitative PCR analysis. Additional file 2: Figure S1. Brain sections from SD and HFD-fed WT mice at 2 months stained with Thioflavin S (Green). Sections were counter-stained with DAPI to visualize nuclei. Scale bar = 200 μm. Additional file 3: Figure S2. Zoomed-in images of Iba-1 (red) staining of respective time points from Figure 2d. Nucleus was counter-stained with DAPI (blue). Additional file 4: Figure S3. Iba-1 (red) staining of cortical (A) and hippocampal (C) areas of brains devoid of plaques from WT and APP-Tg mice fed with SD or HFD for 2 months. Iba-1^+^cells were counted from different fields of the cortex (*n* = 20 from two animals) and hippocampus (*n* = 10 from two animals). ** and *** indicate *p* < 0.01 and *p* < 0.001, respectively. (two-way ANOVA with Bonferroni post-test). Additional file 5: Figure S4. Positive pixel count of oil O-red staining in the liver of WT and APP-Tg mice fed with SD or HFD for 1 year. Fixed liver sections were stained with oil O-red and positive pixels (red) were counted using Zen Software. **indicates *p* < 0.01. (two-way ANOVA with Bonferroni post-test, *n* = 2). Additional file 6: Figure S5. Representative images of hippocampal sections from WT mice fed with SD or HFD for 1 year and stained with anti-NeuN (pink), GFAP (gold). Scale bar = 200 μm.

## Abbreviations

AD: Alzheimer’s disease
ApoE: Apolipoprotein E
APP-Tg mice: [B6.Cg- Tg(APPswe,PSEN1dE9)85Dbo/J] mice
HFD: High-fat diet
H&E: Hematoxylin and eosin
NAFLD: Non-alcoholic fatty liver disease
SD: Standard diet
WT: Wild-type, low-density lipoprotein-1 (LRP-1)
