# Supplementary material for: Non-alcoholic fatty liver disease induces signs of Alzheimer’s disease (AD) in wild-type mice and accelerates pathological signs of AD in an AD model
Source: J Neuroinflammation. 2016 Jan 5;13:1. doi: 10.1186/s12974-015-0467-5 (PMC4700622; doi:10.1186/s12974-015-0467-5)
Supplement: Additional file 1: Table S1. — Sequences of primers used for quantitative PCR analysis. [file 12974_2015_467_MOESM1_ESM.docx]

**Supplementary Table 1. Sequences of primers used for quantitative PCR analysis.**

APOE F 5’ – CAG AGC TCC CAA GTC ACA CA – 3’

APOE R 5’ – AGT CGG TTG CGT AGA TCC TC – 3’

TLR1 F 5’ - GTC TCC CCA CTT CAT CCA GA – 3’

TLR1 R 5’ – GCT TGT TCT TCT CTG TGG GC – 3’

TLR2 F 5’ – CAT CTG GAG AAC TCT GAC CC – 3’

TLR2 R 5’ – CAA AGA GCC TGA AGT GGG AG – 3’

TLR6 F 5’ – ACA CAA TCG GTT GCA AAA CA – 3’

TLR6 R 5’ – GGA AAG TCA GCT TCG TCA GG – 3’

IL-17A F 5’ – GAA GCT CAG TGC CGC CA – 3’

IL-17A R 5’ – TTC ATG TGG TGG TCC AGC TTT – 3’

IL-6 F 5’ - CGT GGA AAT GAG AAA AGA GTT GTC – 3’

IL-6 R 5’ - TGC TTA GGC ATA ACG CAC TAG GT – 3’

LRP1 F 5′ - GACCAGGTGTTGGACACAGATG - 3′

LRP1 R 5′ - AGTCGTTGTCTCCGTCACACTTC - 3′

IL-1B F 5’ - CCT TCC AGG ATG AGG ACA TGA – 3’

IL-1B R 5’ - TGA GTC ACA GAG GAT GGG CTC – 3’
