# Supplementary figures and images for: Non-alcoholic fatty liver disease induces signs of Alzheimer’s disease (AD) in wild-type mice and accelerates pathological signs of AD in an AD model
Source: J Neuroinflammation. 2016 Jan 5;13:1. doi: 10.1186/s12974-015-0467-5 (PMC4700622; doi:10.1186/s12974-015-0467-5)

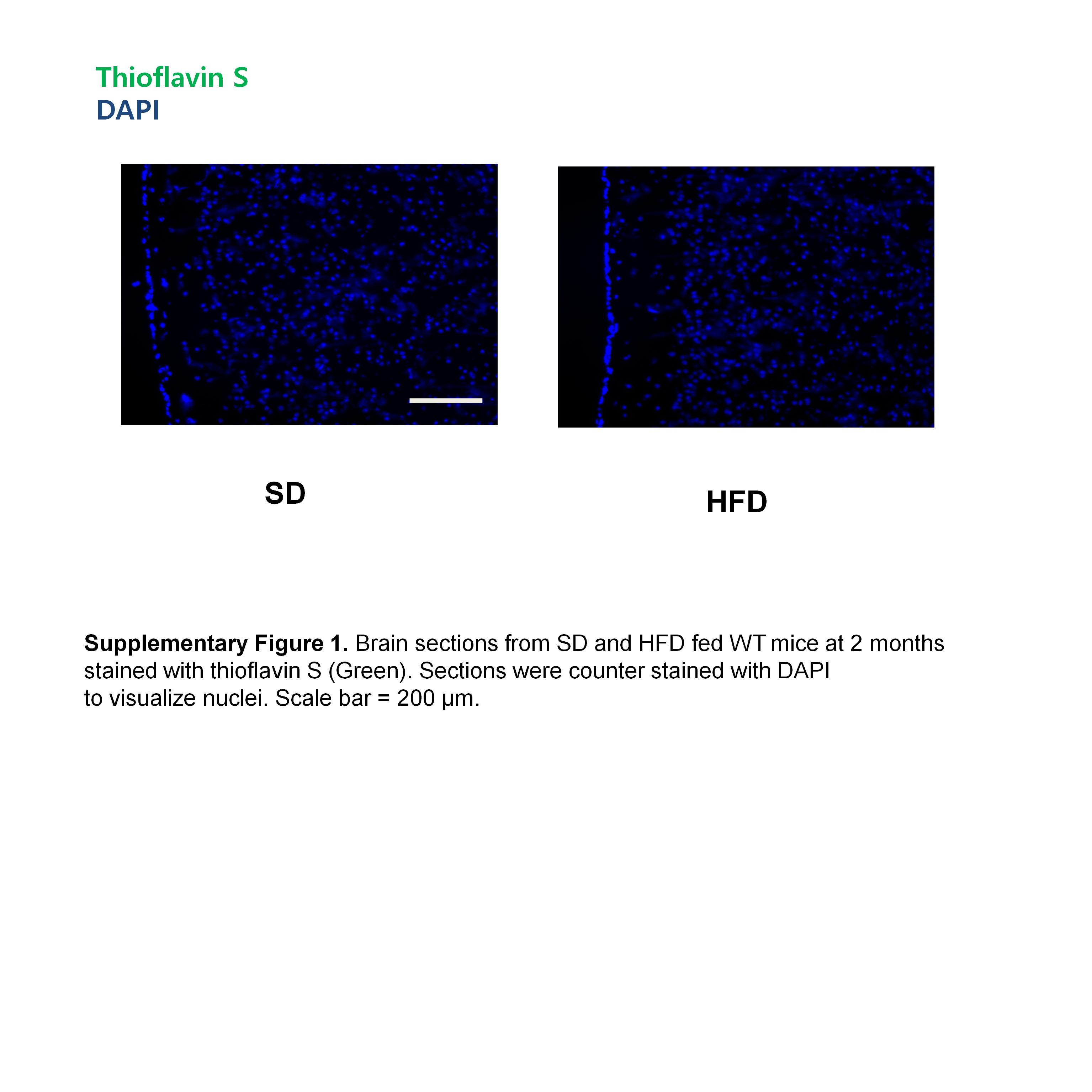

Supplement: Additional file 2: Figure S1. — Brain sections from SD and HFD-fed WT mice at 2 months stained with Thioflavin S (Green). Sections were counter-stained with DAPI to visualize nuclei. Scale bar = 200 μm. [file 12974_2015_467_MOESM2_ESM.tif]

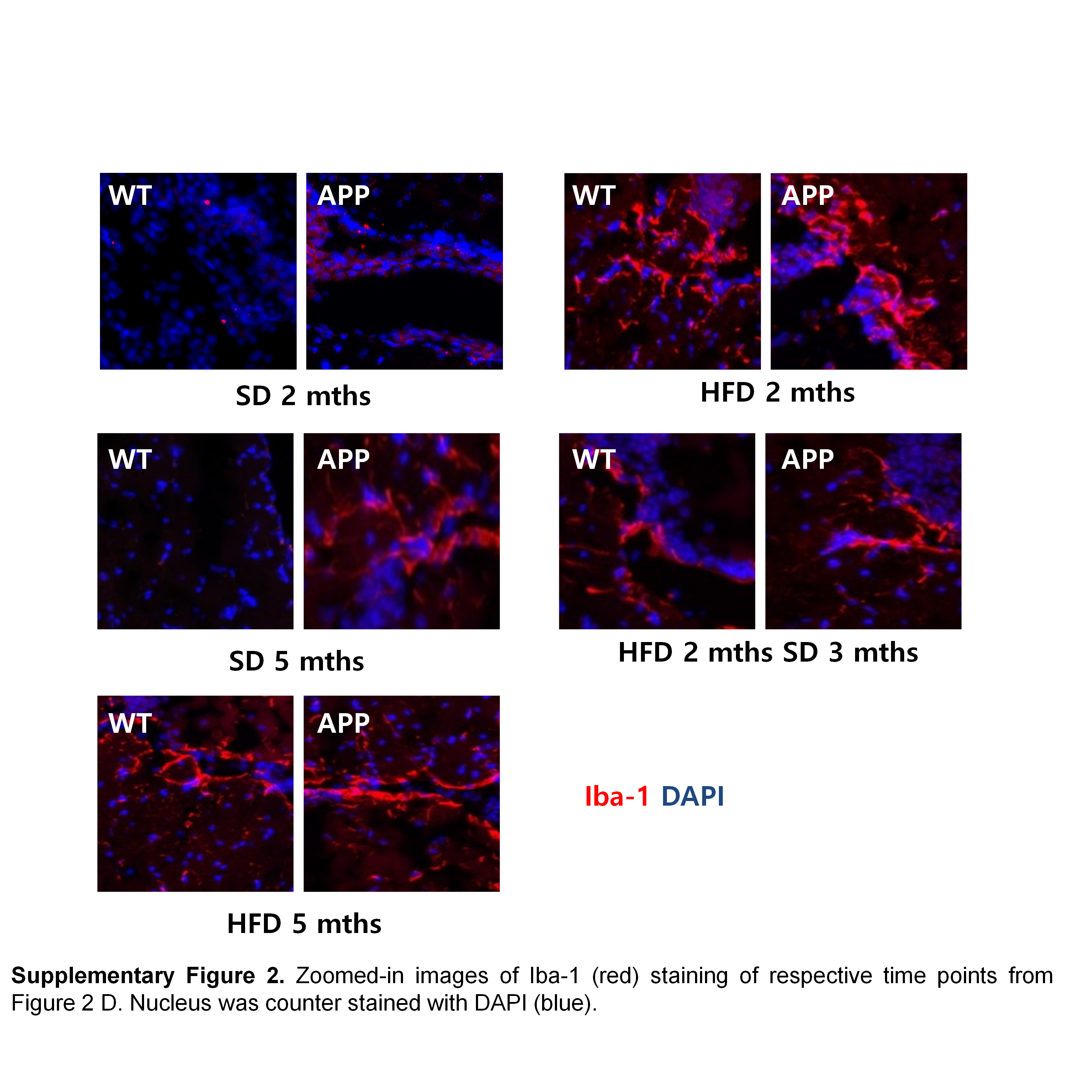

Supplement: Additional file 3: Figure S2. — Zoomed-in images of Iba-1 (red) staining of respective time points from Figure 2d. Nucleus was counter-stained with DAPI (blue). [file 12974_2015_467_MOESM3_ESM.tif]

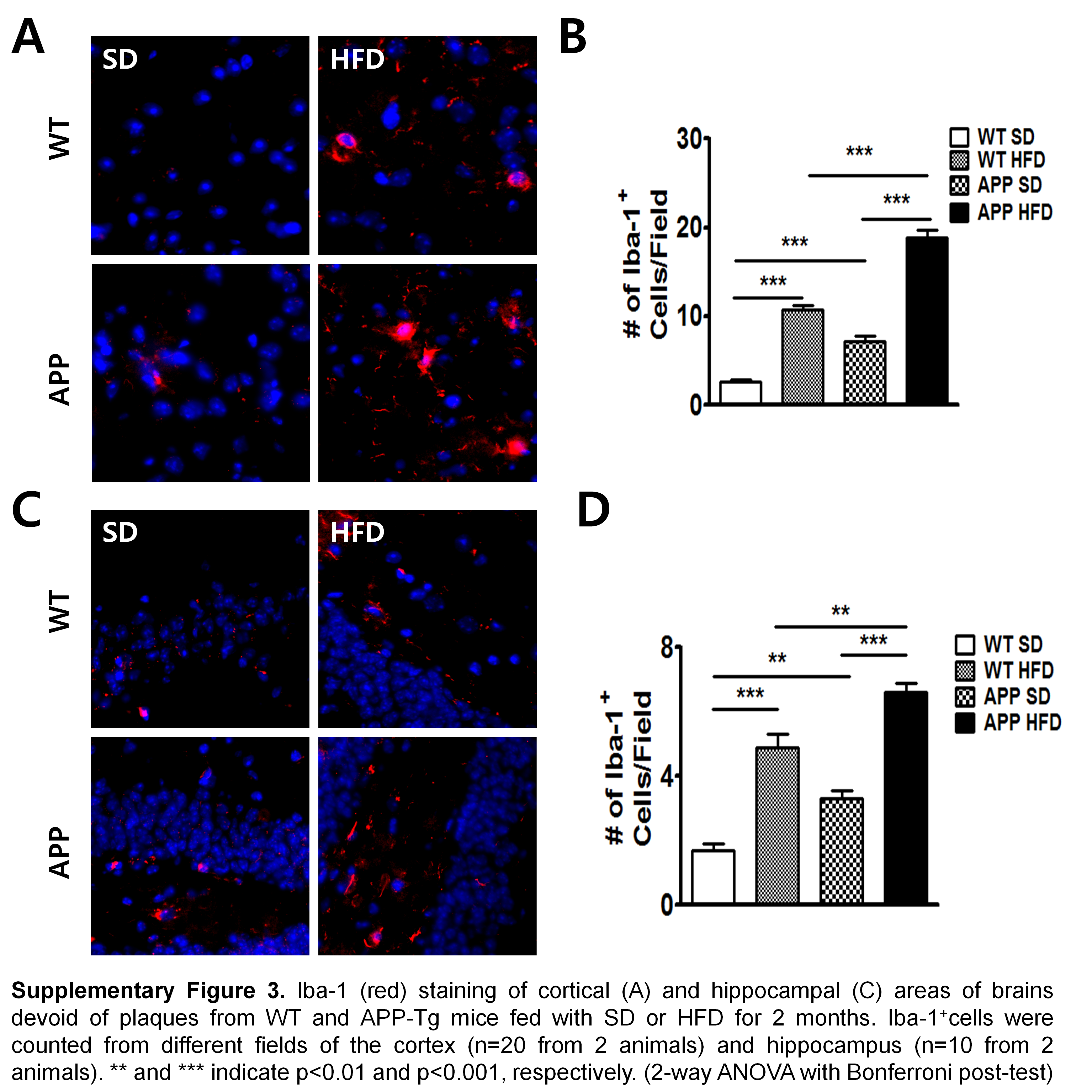

Supplement: Additional file 4: Figure S3. — Iba-1 (red) staining of cortical (A) and hippocampal (C) areas of brains devoid of plaques from WT and APP-Tg mice fed with SD or HFD for 2 months. Iba-1+cells were counted from different fields of the cortex (n = 20 from two animals) and hippocampus (n = 10 from two animals). ** and *** indicate p < 0.01 and p < 0.001, respectively. (two-way ANOVA with Bonferroni post-test). [file 12974_2015_467_MOESM4_ESM.tif]

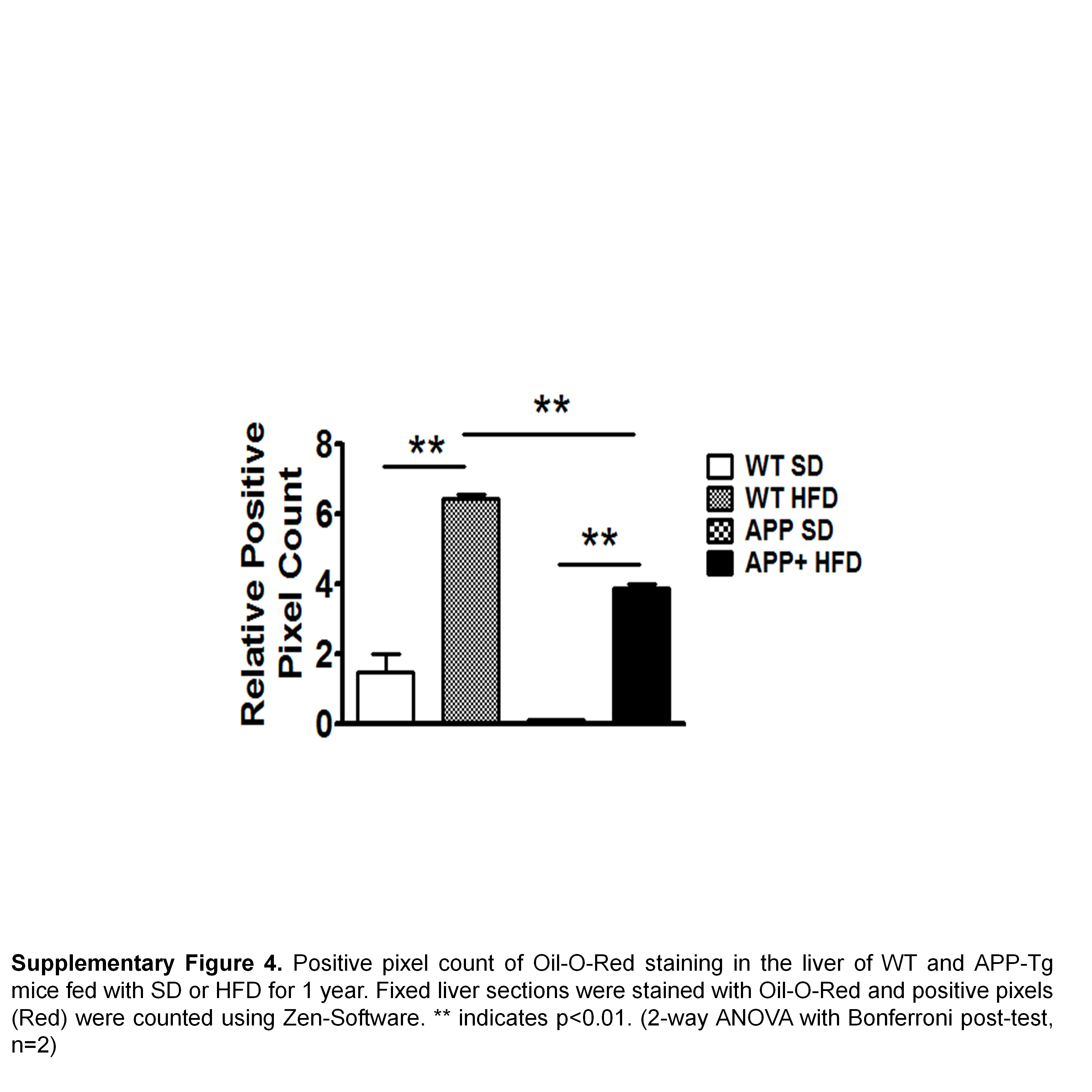

Supplement: Additional file 5: Figure S4. — Positive pixel count of oil O-red staining in the liver of WT and APP-Tg mice fed with SD or HFD for 1 year. Fixed liver sections were stained with oil O-red and positive pixels (red) were counted using Zen Software. **indicates p < 0.01. (two-way ANOVA with Bonferroni post-test, n = 2). [file 12974_2015_467_MOESM5_ESM.tif]

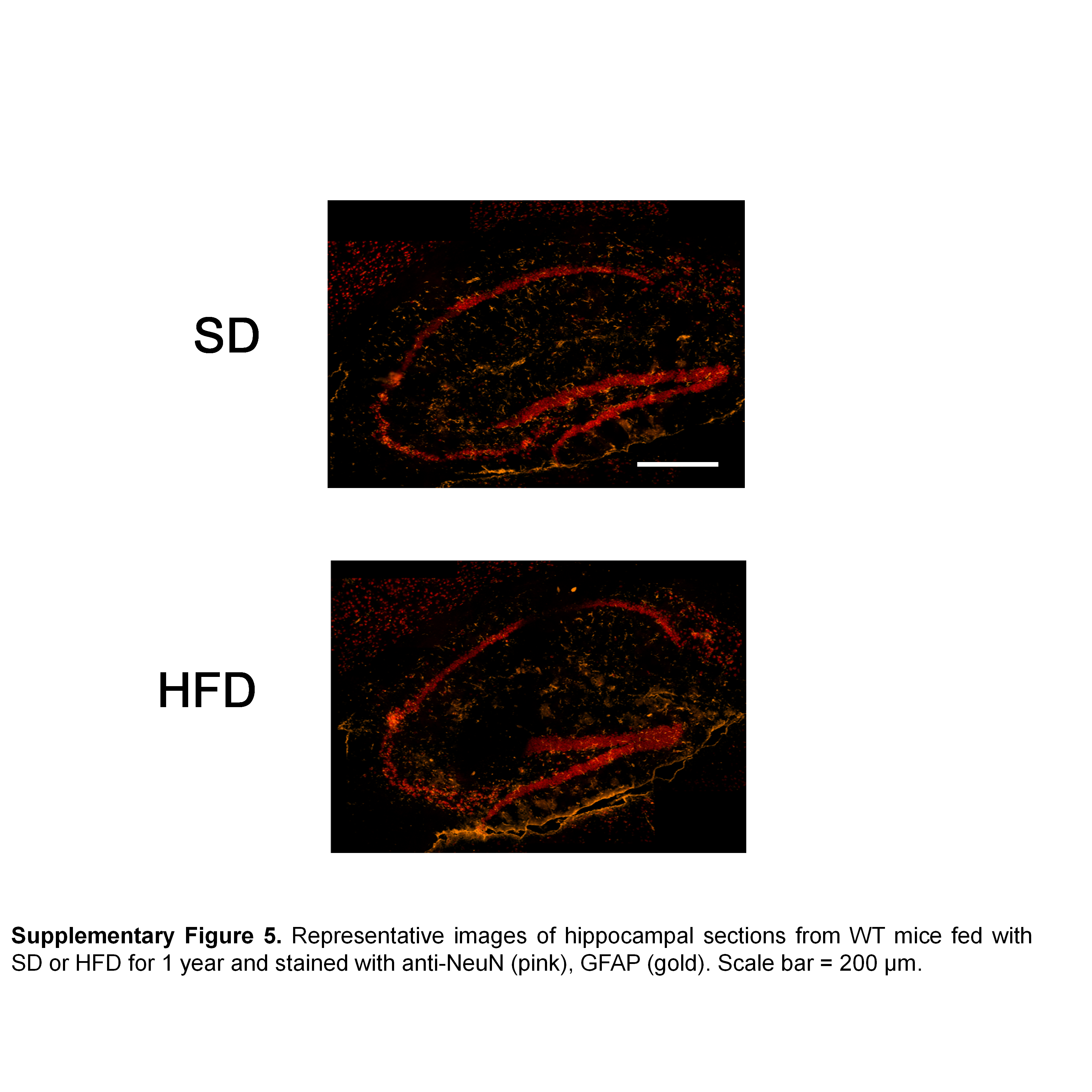

Supplement: Additional file 6: Figure S5. — Representative images of hippocampal sections from WT mice fed with SD or HFD for 1 year and stained with anti-NeuN (pink), GFAP (gold). Scale bar = 200 μm. [file 12974_2015_467_MOESM6_ESM.tif]
